# Priority setting in health care: Lessons from the experiences of eight countries

**DOI:** 10.1186/1475-9276-7-4

**Published:** 2008-01-21

**Authors:** Lindsay M Sabik, Reidar K Lie

**Affiliations:** 1 Department of Philosophy, University of Bergen, Bergen, Norway; 2 Department of Bioethics, Clinical Center, National Institutes of Health, Bethesda, MD, USA

## Abstract

All health care systems face problems of justice and efficiency related to setting priorities for allocating a limited pool of resources to a population. Because many of the central issues are the same in all systems, the United States and other countries can learn from the successes and failures of countries that have explicitly addressed the question of health care priorities.

We review explicit priority setting efforts in Norway, Sweden, Israel, the Netherlands, Denmark, New Zealand, the United Kingdom and the state of Oregon in the US. The approaches used can be divided into those centered on outlining principles versus those that define practices. In order to establish the main lessons from their experiences we consider (1) the process each country used, (2) criteria to judge the success of these efforts, (3) which approaches seem to have met these criteria, and (4) using their successes and failures as a guide, how to proceed in setting priorities. We demonstrate that there is little evidence that establishment of a values framework for priority setting has had any effect on health policy, nor is there evidence that priority setting exercises have led to the envisaged ideal of an open and participatory public involvement in decision making.

## The challenge

One main challenge for health care systems is that resources are limited, making it impossible to provide everyone with every effective intervention they might need or want. Scarcity raises questions of justice and efficiency: how should limited health care resources be allocated? What health services should be publicly funded? How should indications for particular interventions be defined? [1-6].

Priority setting or rationing in health care continues to be a politically charged topic, but recently its necessity has gained wider recognition [7-11]. Explicitly addressing priority setting is necessary to develop fairer methods of allocation for scarce health care resources [7,10,12] and to begin a public dialogue to ensure legitimacy in the process [3,5].

In this paper we will examine seven countries, Israel, Norway, the Netherlands, Sweden, Denmark, New Zealand, and the United Kingdom, and one state in the US, Oregon, that have made explicit efforts to address health priorities. While their systems differ, many core allocation issues are the same [13-21]. The countries vary in how health care is financed (Table 1). Some, such as the UK and the Scandinavian countries, have tax based national health care systems, whereas others, such as the Netherlands, New Zealand and Israel, rely on various forms of universal social insurance. The priority setting approaches can be broadly grouped into two categories: outlining principles and defining practices. In the following we will discuss the efforts under two broad headings. Some countries such as Norway, the Netherlands, Sweden, and Denmark decided to develop principles that would guide prioritization efforts, while others, such as the UK, Israel, New Zealand and the state of Oregon established bodies that would actually recommend what services should be provided within the system. In assessing their efforts we will (1) describe the process each country or state used, (2) suggest two broad criteria to judge the success of these efforts, (3) assess which approaches seem to have met these criteria, and (4) using their successes and failures as a guide, make recommendations for priority setting. In the country descriptions we will focus on the structure of the process, and the principles and values guiding the process. In the evaluation section we will assess the actual impact of the priority setting exercises.

**Table 1:** Health expenditure data for 2003*

**Country**	**Health expenditure as percent of gross domestic product**	**Percent of total health expenditure that is government funded**	**Percent of private health expenditure paid out of pocket**	**Per capita total health expenditure (average exchange rate (US$))**
**Norway**	10.3	83.7	95.40	4,976
**Netherlands**	9.8	62.4	20.80	3,088
**Sweden**	9.4	85.2	92.10	3,149
**Denmark**	9.0	83.0	92.50	3,534
**Israel**	8.9	68.2	89.10	1,514
**New Zealand**	8.1	78.3	72.10	1,618
**UK**	8.0	85.7	76.70	2,428
**US**	15.2	44.6	24.30	5,711

*World Health Organization, Core Health Indicators, from *World Health Statistics 2006*

## Explicit priority setting efforts: the outlining principles approach

Since the late 1980's many governments have instituted transparent and explicit discussions about priorities for health care [14,22]. These efforts took different forms: all included health care experts, but they differed in inclusion of government officials and public representatives (Table 2) and in the details of the frameworks they outlined (Table 3). In most, if not all countries, the priority setting efforts started in response to political reasons to address the issue. In the UK, and to a certain extent the Scandinavian countries, the newspapers continuously reported cases where patients were denied potential life saving treatments, such as bone marrow transplantation for certain cancers. In the UK, in addition, there were reports of differential access in different parts of the country, labeled rationing by post-code. In Norway, the ever-expanding waiting lists for treatment created political pressure for a system that would prioritize patients on waiting lists. In countries such as the Netherlands and Israel, new legislation regarding health insurance created a need to decide what services should be provided in the package offered to all citizens. In Oregon, although Medicaid would provide expensive treatments for all those covered by the scheme, not everyone was covered, and the effort was launched in part to eliminate high cost, low effectiveness interventions and use the subsequent savings to increase the number of people covered by Medicaid.

**Table 2:** Processes for priority setting discussions

**Country**	**Government officials included**	**Health professionals included**	**Public/lay representatives included**	**Government commission**
**Denmark**	NO	YES	YES	YES
**Israel**	YES	YES	YES	YES‡
**Netherlands**	NO	YES	NO	YES
**New Zealand**	NO	YES	NO†	YES‡
**Norway**	YES	YES	YES*	YES
**Sweden**	NO§	YES	YES§	YES
**UK**	NO	YES	YES	NO
**Oregon (US)**	YES	YES	YES	YES

*patient representatives.

† indirectly, through public meetings and surveys.

‡ regular review process.

§included members of parliament

**Table 3:** Overview of centralized priority setting efforts

**Country**	**Principles, guidelines, recommendations**
Year	
Process	
**Norway**	Priority principles:
1987/1997 Lønning Committee I and II	• Severity
	• Potential effect
	• Cost-effectiveness
	Priority groups based on severity (and later funding):
	• Fundamental
	• Supplementary
	• Low priority
	• No public funding

**The Netherlands**	Sieves/filters to determine basic package of services:
1992/1995 Dutch Committee on Choices in Health Care (Dunning Committee)	• Is care necessary?
	• Is care efficient?
	• Is care effective?
	• Can care be left up to individual responsibility?

**Sweden**	Ethical platform principles:
1993/1995 Commission of Parliament members and experts	• Human dignity
	• Need and solidarity
	• Cost-efficiency
	Political/administrative and clinical priority groups:
	• Life-threatening acute diseases, severe chronic diseases and palliative terminal care
	• Prevention and habilitation/rehabilitation
	• Less severe acute diseases
	• Borderline cases
	• Care for reasons other than disease

**Denmark**	Core values:
1997 Danish Council of Ethics	• Equal human worth
	• Solidarity
	• Security and safety
	• Freedom and self-determination
	General goal, framed in terms of "opportunity for self-expression...irrespective of social background and economic ability"
	Partial goals:
	• Social and geographical equity
	• Quality
	• Cost-effectiveness
	• Democracy and consumer influence

**Israel**	Criteria for prioritization of recommended technologies:
1995 Medical Technology Forum and National Advisory Committee, In response to new National Health Insurance Law	• Life-saving technology with full recovery
	• Potential to prevent mortality or morbidity
	• Number of patients to benefit
	• Financial burden on society and the patient
	• New technology for diseases with no alternative treatments available
	• Brings increase in longevity and quality of life
	• Benefits of reducing morbidity vs. improving quality of life
	• Net gain is higher than short- or long-term cost
	• Funding of efficacious treatment than is expensive to the individual, but of reasonable cost to society

**New Zealand**	Set out principles to guide priority setting decisions:
Yearly, beginning in 1993 Core Services Committee/National Health Committee	• Effectiveness
	• Efficiency
	• Equity
	• Acceptability
	'Consensus conferences' for specialized services;
	Recommend core services for given year

**Oregon (US)**	Developed Quality of Well-Being Scale;
Beginning in 1989 Health Services Commission	Used scale to establish cost-effectiveness rankings;
	Revised rankings after public backlash;
	Continued to use ranked list of condition-treatment pairs
	Recently more emphasis on evidence base for recommendations

**UK**	Appraisal of new health technologies;
Ongoing, beginning in 1999 National Institute for Clinical Excellence (NICE)	Development of clinical guidelines;
	Explicit use of cost-effectiveness evaluations
	Appeal possible on narrow grounds

### Norway

In 1987, in the context of increased demand for health care resources and the question of how to prioritize their use, the Norwegian government convened the Lønning Commission, the first body to set forth principles for prioritization and discuss their implementation[23,24]. The commission decided to use severity of condition as the exclusive basis for prioritization, outlining five priority groups

▪ Emergency care for life-threatening diseases

▪ Treatment which prevents catastrophic or very serious long-term consequences: example, cancer

▪ Treatment which prevents less serious long term consequences: example, hypertension

▪ Treatment with some beneficial effects: example, common cold

▪ Treatment with no documented effects

They believed this division could guide funding decisions for various treatments [24]. Ten years after the first, Norway convened a second commission. This commission acknowledged the need to take into account potential effect and cost-effectiveness as secondary principles to be balanced with severity, and introduced four priority groups: core or fundamental services, supplementary services, low priority services, and services with no priority. The commission attempted to define what services should be included in the first category by providing three criteria:

▪ Risk of dying from disease within five years is more than 5–10% (severity)

▪ Increase in probability of five year survival is greater than 10% (expected treatment outcome)

▪ Costs reasonable in relation to benefits

This second commission focused more on the process of setting priorities, than on the principles used to set them [23,25]. One of their suggestions was that the process of priority setting should be started by establishing clinician groups that would set priorities within their specialties.

### Netherlands

In 1990, the Netherlands established the Committee on Choices in Health Care, the so-called Dunning Committee, to discuss methods and principles for setting priorities. That year, the Dutch Cabinet decided to include approximately 95% of available health services in the publicly funded package [26,27]. The committee felt that to ensure all necessary services could be readily provided, non-essential services should be eliminated from the package. It delineated four priority principles: necessity, effectiveness, efficiency, and individual responsibility. They described these principles as forming a sieve for sifting out services that should not be publicly funded ("Dunning's funnel") [27]. The idea was that one should apply these successively beginning with the principle of necessity, and then limiting the number of services provided. The principle of necessity was defined very broadly, basically meaning any intervention that could provide some medical benefit. With regard to the principle of effectiveness, the committee distinguished between interventions where there was evidence for an effect, where there was limited evidence, and where there was no evidence. The services would be further narrowed down by those that gave value for money, by only funding efficient services, and finally, services that were best dealt with by the individuals themselves were excluded. This latter criterion was not meant to exclude lifestyle choices, such as illness because of bad eating habits. Rather, it was meant to exclude services that could easily be paid for by the individuals themselves. One example the committee gave was routine adult dental care. Although such dental care is necessary, effective and efficient, adults can easily pay for such services out of pocket, and it is therefore best left to an individual's responsibility. There was also a strong focus on solidarity and emphasis on approaching macro-level decisions from the community point of view. While Dutch attitudes seemed to be shifting to accept more reliance on personal responsibility, the Dunning Committee did not want changes based on this shift to overlook the needs of vulnerable populations [26].

### Sweden

In 1992, Sweden convened the Parliamentary Priorities Commission. Much work on substantive issues was left to local health authorities; however the central government commission outlined three platform principles: human dignity, need and solidarity, and cost-efficiency. The commission defined five priority groups based on the type of disease or treatment in question [17]. While cost-efficiency was listed as a platform principle, the committee was clear that cost should only be considered in comparing treatments for the same condition, and that measures of effectiveness that attempt to quantify quality of life, such as quality adjusted life years (QALYs), should not be used. As in the Netherlands, there was an emphasis on solidarity and needs of vulnerable populations. In 1994, Sweden convened a second committee, which focused more on eliciting public opinions, a factor that had virtually no role in the work of the first commission [21].

### Denmark

In 1996 the Danish Council of Ethics laid out values that should form the basis of the health service: equality, solidarity, security, and autonomy. From these values, the council derived a general goal for the health service, framed in terms of "opportunity for self-expression...irrespective of...social background and economic ability" [28]. In attempting to fulfill this general goal, there were a number of "partial goals" under consideration, including equity, quality, cost-effectiveness, and democracy. The committee was explicit that these secondary considerations must be "balanced against each other," and that even once goals are agreed upon it can "become difficult when these goals and partial goals are to be translated into decisions with consequences for everyday life in the health service" [28]. However, it did not specify methods for choosing between them.

## Explicit priority setting efforts: the defining practices approach

Rather than begin with abstract principles, Israel, New Zealand, the UK and the state of Oregon confronted priority setting in the context of concrete allocation decisions, such as defining a package of publicly funded services or establishing clinical guidelines.

### Oregon

The experience of priority setting in Oregon's Medicaid program starting in the early 1990's represents the most explicit, as well as one of the most controversial, examples of priority setting in health care in the US. The goal of the program was to extend coverage to all Oregonians below 100% of the federal poverty level (as opposed to 58% FPL, as it was previously) by limiting coverage to a basic bundle of services decided by the Medicaid budget and a cost-effectiveness ranking of available medical services [29-32]. The Oregon process was concerned with incorporating public values from the beginning, and the Oregon Health Services Commission sponsored eleven public hearings, forty-seven town meetings and fielded a telephone survey before the initial rankings were decided [29]. The information gathered was employed in developing the Quality-of Well-Being Scale used to determine the cost-effectiveness ranking of condition treatment pairs and subsequently the services that would be covered by Medicaid [33]. This initial method was, however, abandoned because of public outcry over the resulting ranking of services. In response to this public reaction as well as criticism of the methodology, the Commission basically set out to rank health care services based on more broadly defined criteria, where expert knowledge and intuitive judgments by the Commissioners about appropriateness played a much larger role. While the program of rationing Medicaid services in Oregon was successful in covering a larger population and reducing the number of uninsured, it was extremely controversial and faced a number of political and practical roadblocks along the way [32]. The Oregon Health Services Commission continues to this day and continues to update the methodology used to prioritize and the resulting list of prioritized services. There are several community representatives on this Commission, although its ongoing work is not accompanied by as much public discussion as was the original implementation of the program. Oregon presents an example of both the potential and difficulty of implementing explicit priority setting in the US context.

### New Zealand

In 1993, New Zealand established its first National Advisory Committee on Core Health and Disability Support Services to evaluate which services should be included in the publicly funded health package. While recognizing that existing practices represented an ad hoc list of priorities [34], the Core Services Committee started with this list and worked to identify: 1) discrepancies in provision and management of services (between Maori and non-Maori, men and women, urban and non-urban populations, populations in different geographic regions, etc.), 2) areas where efficiency could be improved, and 3) preferences of communities regarding health care.

While the committee in New Zealand discussed principles in much the same way as other commissions, the discussion occurred in the context of making recommendations about covered services. The committee rejected the idea of having an "Oregon-like" list of services constitute the basic package, but did define eligibility criteria for coverage of specific services. In order to make appropriate recommendations, the Committee looked at unit cost and volume of treatment data for common conditions and identified areas where efficiency could be improved [34,35]. They also used information on public values and opinions, gathered through public meetings, to inform their recommendations. Subsequently, the Committee, renamed the National Health Committee (NHC) in 1996, met yearly to reevaluate and recommend changes to publicly funded health services based on new information or developments.

### Israel

In 1995 Israel passed a National Health Insurance (NHI) Law, guaranteeing health insurance coverage to all citizens. The insurance would be provided by competing private sick funds, with the government acting as the single payer. Under the NHI law, all of the sick funds are required to provide the same basic basket of services to enrollees, with services not in the basic basket available at additional cost to the individual. When the law was first adopted it was decided that the basket comprising the extensive list of services offered by the largest existing sick fund at that time would also be the basic basket covered under the law. Thus, as in New Zealand, no explicit process for deciding on the basic basket was undertaken at the time, though it was recognized that a process was needed for updating the basic basket of services [36,37].

The Ministry of Health created a process to undertake technology assessment and make recommendations regarding the updating of the basic basket of services. Under this system new technologies are screened on a regular basis in order to identify those with no existing alternative or with significant clinical efficacy compared with existing technologies. The assessment of these technologies includes the "evaluation of evidence-based clinical, epidemiological and economic aspects" [[38], p 172]. After several ad-hoc teams assess these aspects of the identified set of new technologies, their evaluations are passed on to the Medical Technology Forum, chaired by the Director of Medical Technology and including senior officials, managers, and researchers in technology assessment. Using a set of guiding criteria (including considerations such as the potential of the technology to prevent mortality or morbidity, the number of patients to benefit, the financial burden on society and the individual patient, and whether the net gain to society is higher than the cost) the Forum grades each technology on a scale of 1–10 and categorizes each as high, intermediate, or low priority. The rankings are then passed on to a National Advisory Committee, which, as of 1999, includes public representatives in addition to officials from the Ministries of Health and Finance and the sick funds. The Committee takes into account the assessments and recommends whether new technologies should be included in the basic basket, as well as limitations on their use [38].

### United Kingdom

From its early days, the idea of rationing was a contentious political issue in the British National Health Service (NHS) [15,39]. When other countries began convening commissions on priority setting there was a call by some for the UK to follow [19,40-43]. Instead, in 1999 the National Institute for Clinical Excellence (now the National Institute for Health and Clinical Excellence) (NICE) was established to: 1) appraise new health technologies, 2) develop clinical guidelines, and 3) assess interventional procedures [44,45]. In conducting these activities, NICE addresses questions including what constitutes necessary and appropriate care, how to incorporate cost-effectiveness considerations, and what interventions should be publicly funded. NICE also includes various pathways for public input.

## Criteria for evaluating priority setting efforts

How well did these priority setting efforts succeed? We propose three criteria for evaluating these efforts. The first criterion is adequate public input in the priority setting exercise. The second criterion is appropriate principles, including the incorporation of an evaluation of the costs and benefits of interventions. The third criterion concerns the effect of the prioritization effort: has it actually had an impact on policy and practice, including the establishment of a review process to evaluate performance (Table 4). Let us briefly justify the selection of these criteria.

**Table 4:** Criteria by which to judge priority setting efforts

**(1) *Public input and discussion***	solicit public input in order to inform health professionals and policy makers about the beliefs, opinions, and preferences of the public. promote a public discussion which aims to educate the public about the need for and options for setting priorities
**(2) *Appropriate principles***	establish a coherent, specific and action guiding set of publicly acceptable principles on which to base priorities, including a practically useful and balanced method for incorporating cost into the prioritization process
**(3) *Effect on policy and practice***	exhibit a sustained effect on the policy and practice of health care priority setting, including through the establishment of an iterative process for review, evaluation, and reconsideration of priority setting determinations

It has often been stressed in the prioritization literature that it is necessary to engage the public in order to gain acceptance for the often painful choices that need to be made. This can be achieved through different mechanisms, including, for example, group exercises in choosing hypothetical health care packages [3,46-48]. Not only is it necessary to educate the public about the need for prioritization, it is also generally accepted that the public should have a real influence on how these choices are made. Most commentators reject an approach where these decisions are made by technocrats behind closed doors without public input. While it can be difficult to realize, public engagement with prioritization issues is necessary to ensure fairness and legitimacy [5,10,49-51] Exactly how this is done can vary. One may simply attempt to elicit the views of the public and incorporate these into decision making [52], or one may aim at a more deliberative process where one arrives at a consensus after a public dialogue. Still unresolved are the questions of how extensive public involvement in the priority setting process should be and who best represents the public's views. There are also problems of ensuring that avenues for public input established on paper are implemented and that input reflects broad and relevant community views [52-55].

Principles and procedures are supposed to help ensure prioritization is consistent with society's values and goals for the health care system. In fact, most commissions were established precisely to establish shared values on which prioritization decisions can be based. Producing value for money is central to efficiently allocating health care resources and will likely lead to fairer allocation as well. We have therefore noted specifically how the commissions have dealt with the issue of cost. A successful approach will integrate cost considerations, rather than acknowledging but avoiding the task of directly addressing the issue. Despite its limitations, CEA is currently the best developed and most used approach to assess whether interventions produce value for money [56-62].

Discussions about how to fairly and efficiently set priorities serve little purpose if they do not impact how priorities are established. Hence, exercises in priority setting must be concretely linked to policy and practice, and we expect that the work of government commissions will have some influence on what interventions are covered. In some cases, such as for NICE in the UK, the bodies have a direct influence on coverage decisions, and are set up to influence health policy. For commissions given the task of establishing a framework for priority setting, the influence will have to be more indirect. One important result would be if the work has led to an increased awareness among the general public about the need for priority setting, as we noted above. Another result would be if some of the concrete recommendations by the commission were subsequently adopted. Because of the controversial nature of most prioritization decisions, it is particularly important to note what procedures have been established for review and appeal of decisions reached.

## Evaluating the eight efforts on priority setting

The approaches of the eight countries we consider vary in how well they fulfilled these criteria.

### Solicitation of public input and promotion of public discussion and education

To fully evaluate the extent to which the priority setting bodies have promoted public discussion and education, we would have to distinguish between what the commissions did to encourage discussion during their tenure, and any effects the commissions have had on subsequent public involvement. Here we will confine ourselves to the involvement during the existence of the commissions, and the structures set up by the standing bodies established. All the priority setting bodies recognized the importance of transparency in setting priorities, by emphasizing the importance of promoting public discussion in order to make the need for priority setting clear. The Swedish commission, for example, emphasized that public discussions of priorities help clarify the reasons and methods on which priority setting decisions are based [21]. Similarly, the Dutch Committee emphasized the importance of introducing the topic of priorities into public discussion, not only to make people aware of the need for prioritization, but to encourage individuals to make their own choices regarding health care options [27]. The Commissions, however, varied in the way the public was involved.

While Norway involved public representatives on its commissions, the Norwegian Commissions only discussed the importance of public education, whereas the Danish Council actually held public meetings and distributed materials on priority setting [28]. The Commissions in the Netherlands and Sweden actively incorporated feedback from public meetings and surveys into their deliberations [21,27]. The second Swedish commission referred to four public surveys, two of which it funded, and held five regional conferences[21,63]. The Dutch committee set out a plan for a long-term program to solicit feedback from various consumer groups, including women, the elderly, the "low-involved," and different patient groups by beginning a discussion primarily through the media, then soliciting feedback through various meetings [27].

One of the stated goals of the New Zealand's Core Services Committee was to ensure that core health services "reflect the diverse needs and values of the population being served" [34]. The first step in this effort is to inform the public and engage them in a discussion of health services. The New Zealand Committee held periodic "Best of Health" public meetings based around discussion documents. The views expressed at these meetings were part of the committee's considerations in making recommendations regarding core services [64]. Further, they recommended the continuation of consultation by the Health Authorities in charge of funding services with communities, including Maori communities, in different regions [34,65,66]. The NICE model of public involvement allows for public input at different levels on both broad principles and specific guideline development. Input is solicited by including lay members with relevant experience on guideline committees, posting draft guidelines on NICE's website to solicit public feedback before issuing final guidelines, and convening Citizens Councils, composed of 30 individuals representing the public, to discuss questions such as how to define and evaluate clinical needs for treatment or what role age should play in making clinical decisions [44].

In Israel, explicit public involvement was not taken into account in establishing the terms of the NHI Law in 1995, though the importance of involving members of the public in the subsequent decision-making process about the basic basket was quickly realized. Thus, beginning in 1999, a quarter of the membership of the National Advisory Committee that makes the final recommendation to the Minister of Health regarding which new technologies should be added to the basic basket were public representatives with no medical background [38].

### Establishment of principles

Except the UK, all of the countries considered here started the discussion by establishing some set of principles on which to base priorities [14,66]. In the UK, this discussion has gone on through other avenues, but has not been centralized and systematic [39]. The principles cited by the commissions include a range of medical, philosophical, and economic factors [17,21,27,28,34].

It seems unreasonable to base prioritization on a single principle. Indeed, when the Norwegian Commission attempted to formulate a system based exclusively on severity, it found that important considerations, such as effectiveness of interventions and cost, were excluded and saw the need to add additional principles. With multiple principles, the challenge is determining how to balance them when they conflict. As the Danish committee pointed out, balancing "become [s] difficult when [principles] are to be translated into decisions with consequences for everyday life in the health service" [[28], p 57]. While commissions acknowledged the challenge of balancing, none solved the problems they identified.

In general there is hesitancy to place much weight on CEA due to discomfort and uncertainty in dealing with cost. In Norway, the first commission avoided cost [24], and only after negative responses did it add the secondary principles of potential effect and cost-effectiveness. The Danish committee discussed problems with using cost related analyses, including uncertainty about what measure of utility to use and the need for more information. Rather than addressing these problems, the committee was hesitant to endorse the use of any economic analyses [28]. The initial Swedish report stated that cost should only be a deciding factor when "all else is equal"[21]. In both Denmark and Sweden, the commissions specified that cost should only be considered when comparing treatments for the same illness, such as comparing a titanium hip prosthesis to a less expensive but less durable steel prosthesis [21]. While Israel's process does not include a requirement that cost-effectiveness specifically be taken into account in making decisions, it does include an economic evaluation that considers the overall cost of including a new technology in the basic basket by comparing it with currently available treatments. In a small number of cases, explicit cost-effectiveness or cost-benefit analysis is conducted as well ([38] The Dutch Committee in principle allowed the lack of cost-effectiveness to determine that an intervention should not be covered.

Only New Zealand lists cost-effectiveness as a primary consideration [34]. NICE explicitly integrates cost in every case of guideline development and technology assessment [44,67]. Still, the use of CEA in NICE guidelines has been controversial in the past; its recommendation against using beta-interferon for treatment of multiple sclerosis based on cost-effectiveness grounds, for example, caused an outcry from MS groups, and its final recommendation was controversial among the medical and research communities as well [34,65,68-72].

### Impact on policy and practice

Because of their abstractness, government commissions that outlined principles have had little direct impact on their countries' policies. For example, the Danish Council of Ethics explicitly noted that their partial goals would sometimes conflict, yet did not set out methods for implementing them in these cases [28]. Even when a commission outlined priority groups for use in practice, the guidelines were so broad as to be useless in resolving difficult prioritization questions [17,24,27]. In Norway, for example, the division of priority groups into fundamental, supplementary, low priority and no priority did little to help resolve questions of choice in individual circumstances. Further, the lack of political tension within the Swedish Commission, which included parliamentary representatives of all major parties, was seen by many as a sign that the group avoided controversial issues central to priority setting [23].

After the early rounds of priority setting discussions, principles set forth by the committees had little direct effect on health care planning and provision. According to Berg and van der Grinten, the criteria that make up Dunning's funnel were difficult to implement because of contention about the definition of "necessary" and the difficulty of making macro level judgments about the effectiveness and efficiency in specific cases or whether an intervention can be left up to individual responsibility. Even when the Dutch government took the approach of the Dunning committee seriously, "problematic substantive criteria..., financial considerations...and political pressures ...made it very difficult to remove services from the package" of publicly funded health services [[26], p 124]. In general, governments and planning groups continued to make decisions about coverage based on a host of other factors, including political concerns and media pressure [14,61,73].

After a decade of discussions and repeat performances by some commissions, little progress had been made. Changes in health services after recommendations were issued did not reflect the extensive discussions and proposals put forth by the commissions [73,74]. One study showed evidence that less than half of new medical technologies actually implemented between 1993 and 1997 in Norway fit the Lønning definition of core services [74]. "By 2002, few of the recommendations [of the Lønning Commissions in Norway] had been implemented" [[24], p 2003]. In particular, specific priority setting committees were supposed to issue recommendations for different fields of medicine, but as of 2007 this had not been done. While the discussion of priority setting was successfully started, it was not clear that the government commissions had any significant impact on actual practices at the policy, planning, or clinical levels.

The approaches of Israel, the state of Oregon, New Zealand and the UK affected policy and practice most directly. For example, by making specific recommendations regarding covered services in New Zealand and establishing specific clinical guidelines in the UK, the groups in these countries have anchored the discussion of priorities in concrete policy determinations. Yet, within NICE, there is no systematic review of existing health technologies; thus there is a bias towards reviewing only new technologies. While the UK is a step ahead in affecting practice, there are still improvements to be made in the system.

All the countries that have set up bodies that decide on priorities, and the state of Oregon, have ongoing processes that are conducive to iterative reflection on past successes and failures. In Israel, new technologies are screened and assessed for inclusion in the basic basket of services on a regular basis. Similarly, the ongoing nature of NICE creates opportunities for review and evaluation of the process, though the review has been less systematic than in New Zealand. The yearly reevaluation of the health services and discussion of other health care issues by the NHC in New Zealand is the best example of effective review and evaluation [14,41]. For example, in 1996 the Committee recommended against population screening for prostate cancer using the prostate specific antigen (PSA) test, but recommended the question be kept under review [75]. In 2001 the NHC began a review that culminated in a 2004 report echoing their earlier recommendation against population-wide PSA screening [53,76,77]. Rather than attempting to settle questions of prioritization with one discussion, the New Zealand Committee has established an iterative process that allows priority setting to evolve with medical and political changes.

Finally, one should note that the priority setting efforts had little effect on the political events that actually led to the establishment of the priority setting exercises in the first place. The Norwegian effort did not solve the problem of waiting lists, the Dutch politicians did not use the criteria of the Dunning committee to decide what should be included in the package of health services, and the Oregon effort did not lead to any substantial cost-savings by eliminating interventions from the services provided that could be used to increase the number of people covered by Medicaid [24,26,78].

## Future directions for priority setting

What can we conclude from this examination of eight priority setting efforts? First, the bodies established to recommend or decide prioritized interventions have been relatively successful. They key to ensure impact on policy and practice is therefore to establish bodies with some decision making power on what is actually implemented in the health care system. Although controversial, the policies in the state of Oregon, Israel, New Zealand and the UK, have been largely accepted. Second, and in agreement with an apparent consensus in the literature, formulation by public bodies of abstract priority setting principles will not have much impact on policy. In the words of Søren Holm, "The Danish Council argued that none of the priority-setting systems which had been produced were really operational, and all suffered from one or both of two fatal flaws: .... They were based on a simplistic view about the purpose of the health care system; and/or they did not really give any specific guidance as to how one should prioritize" [[79], p. 31]. None of the Commissions given the task of formulating a principled framework for priority setting has had much impact on health policy in their respective countries. This general negative conclusion has led many commentators, including the Danish Commission as well as the second Norwegian Commission and commentators such as Norman Daniels to advocate a different approach [80]. Again, according to Søren Holm, "If we cannot find rule-based systems which can legitimize the decisions, we will instead have to devise priority-setting processes that can lend legitimacy to the outcome." Holm goes on to quote from the Danish Commission describing such a process

There should be an effort to ensure that decision-makers at different levels be aware – informed – of which priority-setting consequences different decisions entail. The issue is ensuring clearness, the necessary information being available, and that analyses have been executed of which consequences different decisions entail. At the same time the public should also be ensured a higher level of information on which decisions are made at which levels, and which reasons there are for the individual decisions. Such openness is crucial to ensuring that individual decisions can be subjected to criticism and possibly changed on the basis of the public debate. For this reason great importance should also be attached to the health planning in the counties being organized in such a way as to ensure the possibility for common citizens to participate in the decision making process, for instance at hearings and public meetings. [[79], p.34]

Our third conclusion is, however, that such a process, in spite of its attractiveness and in spite of the emphasis placed on public involvement in the prioritization literature, has also not really been implemented in any of the priority setting exercises examined here. The two country commissions mentioned by Holm as proponents of this approach, Norway and Denmark, have not yet, a decade after the reports, implemented any process with even the most rudimentary elements of the public process described above. In fact, the recommendation in Norway was that this process should be expert driven, and not involve much public debate. The processes implemented in countries with actual priority setting bodies, Israel, New Zealand, the UK and the state of Oregon in the US, also do not appear to fit the description of an open and transparent process with public discussion and decisions about the trade-offs that need to be made. New Zealand, Israel and Oregon have largely delegated the decisions about what services should be included in the health care package to a body of experts, with few, if any, possibilities for appeal. NICE in the UK, which perhaps comes closest to the ideal of a process for evaluating new technologies, has developed a structure of public involvement at all levels, and there is a possibility of appealing the decisions reached. However, the public is not engaged in the envisaged debate about what trade-offs need to be made, and how to balance different principles. The basis for appeal is also very narrow; successful appeals can only be made if the committee has made obvious mistakes. The three grounds for appeal are only:

▪ The Institute has failed to act fairly and in accordance with its procedures;

▪ The Institute has prepared guidance which is perverse in the light of the evidence submitted; and

▪ The Institute has exceeded its powers

NICE to a large extent limits itself to examining new technologies for their cost-effectiveness, where the major determinant of the decision reached rests on the examination of technical evidence of proven effect, and the costs of the procedure in relation to a more or less pre-set level of cost-effectiveness. In that respect it resembles more a traditional technology assessment agency, rather than a body with a mandate to involve the public in an open dialogue about the difficult moral choices in health care priority setting. In Israel the National Advisory Committee included about a third of the members without any medical background. In its advisory role, however, the committee largely relied on the expert judgments of the Medical Technology Forum, and the final decision about what to include for reimbursement was reached without any public discussion or involvement.

Against this one might want to argue that the case of Oregon demonstrates that the success of a community led process as opposed to a technocrat led process. The initial ranking based on a mechanical application of cost-benefit calculation was abandoned in favor of judgments by a panel with a sizable proportion of community members. This revised methodology was accepted and has been utilized successfully during the subsequent decade. In spite of this, there are two reasons why Oregon is not a counter-example to the position taken in this paper. First, the Health Services Commission has recently recognized that it is necessary to place a much greater emphasis on the evidence for the effectiveness of interventions and its cost-effectiveness [81]. One can therefore expect that the recommendations by experts will place a much larger role in deciding the prioritized list. Second, although there were a large number of public hearings and input during the initial work of the Commission, the work today is largely carried out without much public debate. Furthermore, the initial discussion probably had more to do with gaining acceptance and legitimacy for the process, than about a public deliberation about the conflicting values and reasons that would initially lead different people to come to different conclusions but then, as a result of the open public debate, result in consensus about the list of priorities [78].

What does this mean for the future direction of health care priority setting? In our view, it suggests that we perhaps should reevaluate the place for some type of expert led model of implicit rationing and priority setting in health care. One the one hand, the experience of the priority setting commissions of countries such as Norway, the Netherlands, and Denmark suggests that we will never reach agreement about an explicit framework for priority setting. Although these countries did not establish any priority setting bodies with decision-making power, the intention was that the recommendations of the commissions should be implemented. As we discussed above, this has not yet happened. On the other hand, the experience of actual priority setting efforts in Israel, New Zealand, Oregon and the UK, suggests that this is best done by a group of experts who consider the evidence for the effectiveness of various interventions, without much public involvement and discussion. The experience in these countries show that such an expert led process may be accepted by the general public. The actual acceptance of the public that we have seen in these countries, could, of course, simply be a reflection of the fact that the public feels powerless to influence the process. Some of the public criticism of the specific decisions made by NICE may be a reflection of that fact. The relative success of this approach also does not mean that there should be no public involvement, nor an absence of appeal processes. All the priority setting bodies examined here involve the public and allow for an appeal of the decisions, but it is quite clear that it is much less than is envisaged by those who advocate open and transparent processes involving the public. The challenge of health care prioritization would therefore seem to be to identify an appropriate balance between an expert led process and a process that emphasizes public involvement in decision making. We recognize that this conclusion is controversial, and goes against much of the thinking in the current prioritization literature, where there is much more emphasis on public involvement. The examination of the prioritization efforts discussed in this paper, however, leads us to conclude that implementation of public discussion and open and transparent deliberative processes has not been achieved. In spite of this, some countries have been able to achieve some degree of public acceptance of actual rationing. In light of this, one of the main challenges for the priority setting field would be propose appropriate levels of public involvement and appeal that is much less extensive than the usual rhetoric suggests, but that still ensures that decisions reached are legitimate.

One key element for appropriate public involvement would probably be transparency in providing reasons for decisions. Even though there may not be much possibility of actually appealing a decision and reversing it, the possibility of public discussion and criticism of justifications for decisions, will in all likelihood influence priority setting bodies. Although such influence is largely indirect, in the long run it will probably be more substantial than the formal ability to directly appeal particular decisions.

## Declaration of competing interests

The author(s) declare that they have no competing interests.

## Authors' contributions

Both authors have been involved in initiating the project, carrying out the background research, and the writing of the article. Both authors have read and approved the final manuscript.
